# An *in Vitro* Twist Fatigue Test of Fabric Stent-Grafts Supported by Z-Stents *vs.* Ringed Stents

**DOI:** 10.3390/ma9020113

**Published:** 2016-02-16

**Authors:** Jing Lin, Robert Guidoin, Jia Du, Lu Wang, Graeham Douglas, Danjie Zhu, Mark Nutley, Lygia Perron, Ze Zhang, Yvan Douville

**Affiliations:** 1 Key Laboratory of Textile Science and Technology of Ministry of Education and College of Textiles, Donghua University, Shanghai 201620, China; jlin@dhu.edu.cn (J.L.); 2140126@mail.dhu.edu.cn (J.D.); zhudans2015@gmail.com (D.Z.); 2 Department of Surgery, Laval University and Axe of Regenerative Medicine, Centre de Recherche du CHU, Québec, QC G1V 0A6, Canada; robert.guidoin@gmail.com (R.G.); lygiaperron@gmail.com (L.P.); ze.zhang@fmed.ulaval.ca (Z.Z.); yvan.douville@fmed.ulaval.ca (Y.D.); 3 Department of Engineering, University of Cambridge, Cambridge CB2 1PZ, UK; grd26@cam.ac.uk; 4 Division of Vascular and Endovascular Surgery and Department of Diagnosis Imaging, Peter Lougheed Health Centre and University of Calgary, Calgary, AB T2N 1N4, Canada; nutleym@gmail.com

**Keywords:** stent-graft, Z-stents, ringed stents, twisting, endotension

## Abstract

Whereas buckling can cause type III endoleaks, long-term twisting of a stent-graft was investigated here as a mechanism leading to type V endoleak or endotension. Two experimental device designs supported with Z-stents having strut angles of 35° or 45° were compared to a ringed control under accelerated twisting. Damage to each device was assessed and compared after different durations of twisting, with focus on damage that may allow leakage. Stent-grafts with 35° Z-stents had the most severe distortion and damage to the graft fabric. The 45° Z-stents caused less fabric damage. However, consistent stretching was still seen around the holes for sutures, which attach the stents to the graft fabric. Larger holes may become channels for fluid percolation through the wall. The ringed stent-graft had the least damage observed. Stent apexes with sharp angles appear to be responsible for major damage to the fabrics. Device manufacturers should consider stent apex angle when designing stent-grafts, and ensure their devices are resistant to twisting.

## 1. Introduction

Endovascular surgery has been remarkable in its achievement of making more and more patients amenable to treatments, throughout the arterial tree [1,2,3,4,5,6]. Fenestrations, branches, chimneys, and periscopes are now widely accepted [7,8,9,10] further to their respective validations [11,12,13,14]. Acute device-related failures, as reported in the early days of endovascular surgery, are now rare [15,16,17,18,19,20,21,22]. Registry-based follow-ups [23,24] and device-retrieval programs (including analysis of explants) [25,26,27] have led the various manufacturers to develop devices that are more favored by surgeons and have more durable stents [28,29,30,31]. Regretfully, the fatigue and deterioration of fabric materials *in vivo* is still poorly understood [32,33,34,35,36]. We have previously shown that long-term buckling of the stent-graft can cause fabric damage at the apexes of sharp-angled Z-stents [37]. The design of such stents has been addressed in the most recent generation of stent-grafts [38,39].

**Figure 1:**
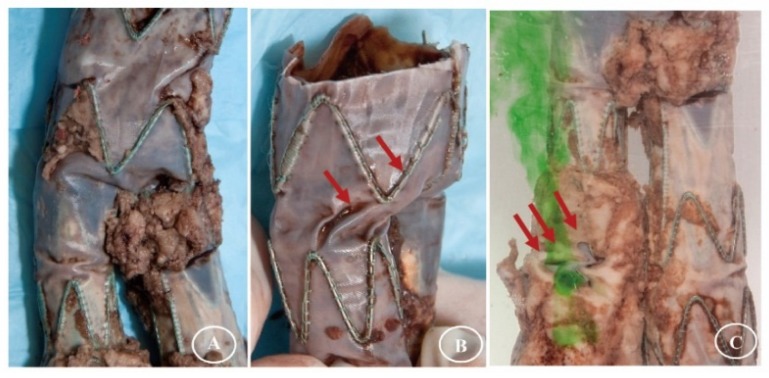
Twisting in explanted Talent stent-grafts. The area of the crotch of the bifurcation holds an irregular encapsulation, likely to prevent blood endoleak (**A**). The twisting can, however, be more evidenced (**B**: arrows) and results in fluid percolation (**C**: arrows).

**Figure 2:**
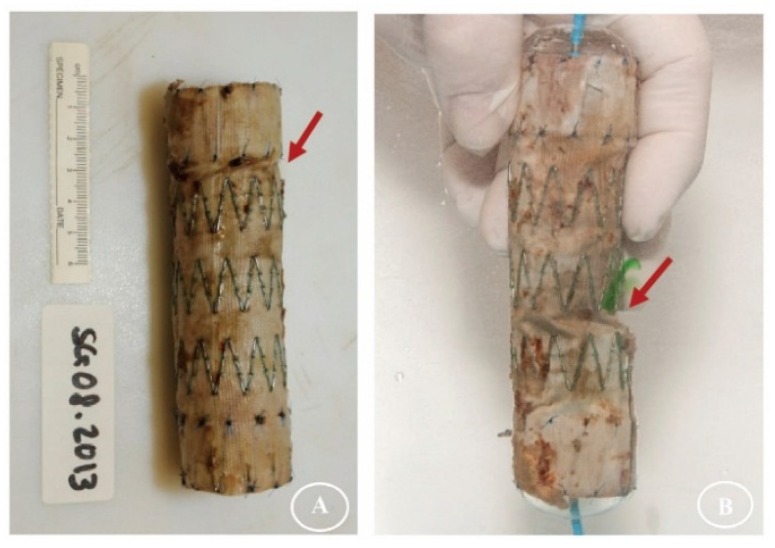
The phenomenon of twisting is well evidenced in an explanted thoracic Zenith stent-graft (**A**: arrows); showing some fluid percolation at the twisting site (**B**: arrows).

**Figure 3:**
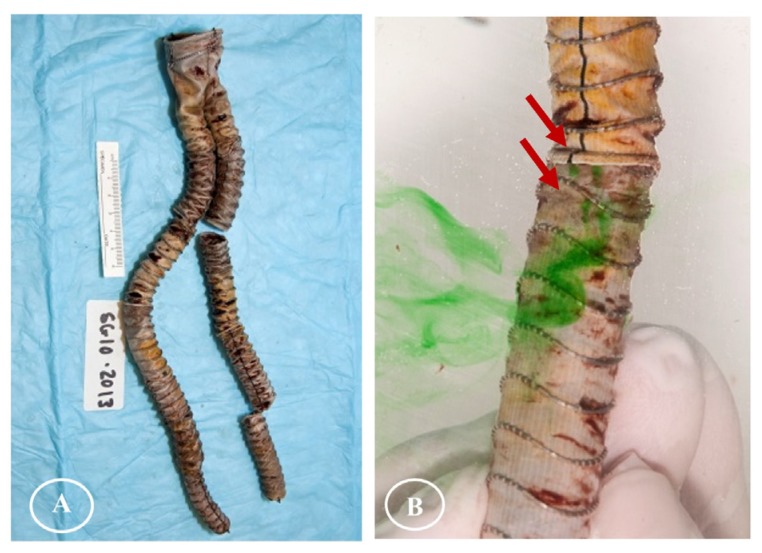
Deformation from twisting in the body of an explanted Anaconda device is maintained after explantation. The fabric preserves its imperviousness according to the gross observation (**A**). However, endoleaks were shown in the distal limb (**B**).

Further, observations in clinical explants of fabric abrasion and perforation, in addition to suture fractures, raised a need for in-depth analysis of the long-term fabric durability [40,41,42,43,44]. Fabrics showed more extensive damage in the areas where the apexes of two adjacent stents may contact. This contact can occur at kinks or buckles in the stent-graft. Additionally, devices deployed in tortuous arteries show various levels of twisting. The development of repositionable stent-grafts has reduced this issue, but has not fully eliminated it [45,46]. Twisting in stent-grafts must be studied because indications of incontinence at the site of the twists have been observed in explants. Explanted commercial Z-stent devices showed percolation when subjected to the periprosthetic endoleak test (Figure 1 and Figure 2). Similar incontinence was observed in the distal limbs of an explanted Anaconda ringed stent-graft (Figure 3).

We hereby report a series of *in vitro* experiments using accelerated testing to understand stent-graft damage from twisting, using devices supported by Z-stents of two different strut angles and ringed stents of one design.

## 2. Results

### 2.1. Gross Observations

**Figure 4:**
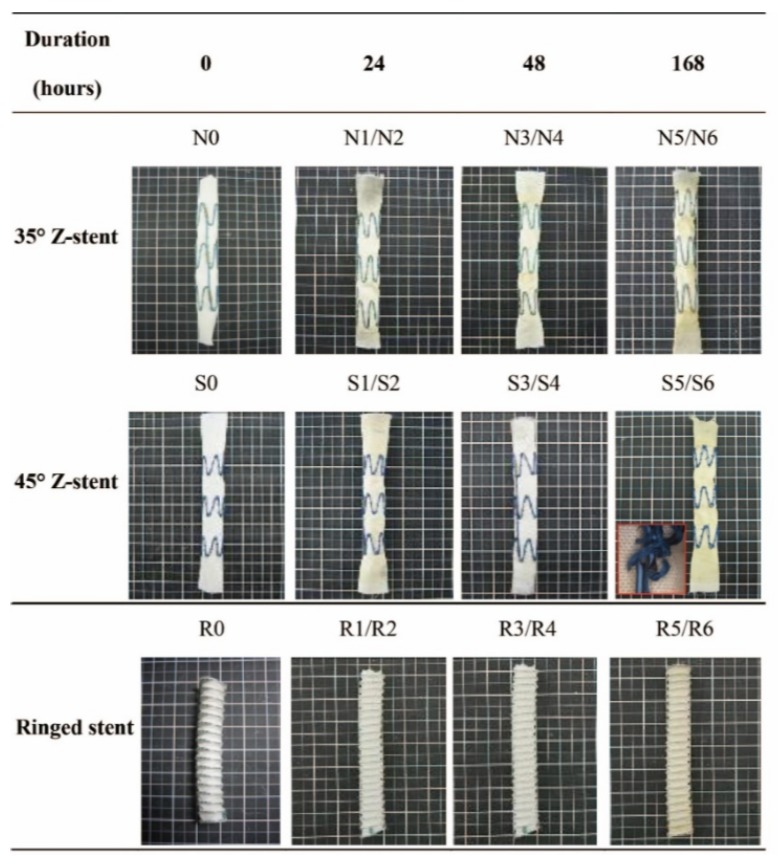
Gross observations after twisting tests. The fabrics of the polyester conduits are without visible holes. However, some sutures are fractured in the Z-stent devices and one vertical strut of a 45° Z-stent is fractured after 168 h of accelerated testing.

After the twisting experiments, the three stent-graft designs had different degrees of damage. No holes were observable in the fabrics without magnification. Some sutures Z-stent devices were ruptured, but the sutures of Anaconda devices (supported by ringed stents) were intact. All the stents remained intact as well, except a vertical strut of S5 (45° Z-stent) was fractured after its 168 h test (Figure 4, S5/S6).

### 2.2. Observations Under Light Microscopy and SEM

**Figure 5:**
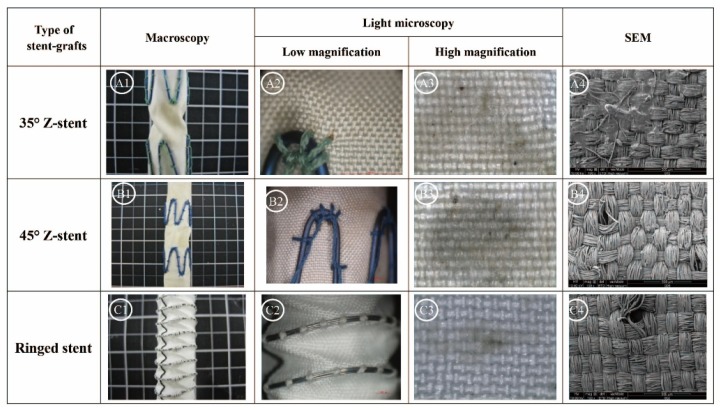
Twist simulations for 24 h. There is no different in damage from fatigue to either Z-stent-graft. Neither stents nor sutures are broken, but the suture holes are enlarged. After the sutures are cut, abrasion is visible at the apex of the 35° stent. The device, stents, and fabric are intact in the ringed stent-graft. (**A1**,**B1**,**C1**) Gross observations; (**A2**,**B2**,**C2**) Observation in light microscopy with low magnification (10×); (**A3**,**B3**,**C3**) Observation in light microscopy with high magnification (40×); (**A4**,**B4**,**C4**) Observation in SEM (100×).

Figure 4 summarizes the gross observations of damage after 24 h of simulated twisting fatigue on the three designs of stent-grafts. There was no difference in fatigue performance of either Z-stent design. Stent-grafts constructed with Z-stents (35° and 45°) appeared to have more fabric kinking and distortion from the twisting compared to those supported with the ringed stents (Figure 5, A1, B1 and C1). While the stents and sutures were intact, the sutures were stretched in the stent-grafts supported by Z-stents (Figure 5, A2 and B2). After removing the stents and sutures from the fabrics, twist abrasion was seen in the fabrics in the vicinity of the apexes of the Z-stents and ringed stents (Figure 5, A3, B3 and C3). SEM confirmed that the fabrics supported by 35° Z-stents were most severely damaged. In comparison, the fabrics supported by ringed stents were better preserved. Enlarged suture holes were observed in all devices after the sutures were removed (Figure 5, A4, B4 and C4).

**Figure 6:**
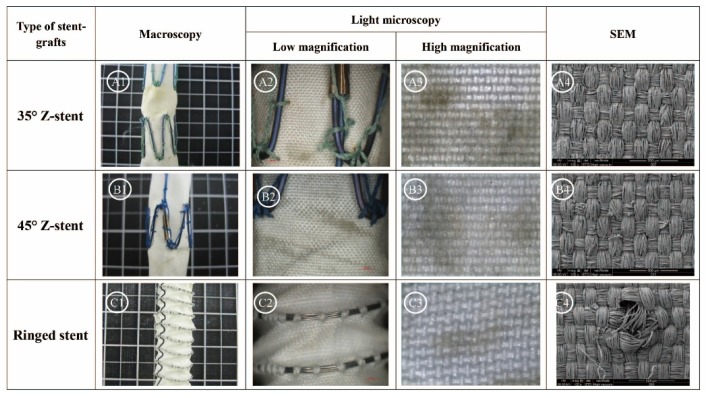
Twist simulations for 48 h. The fabrics of the stent-grafts supported by Z-stents were more severely abraded than the fabric of the ringed stent-graft. Broken sutures are found in the 35° Z-stents. The SEM confirms the best resistance to fabric abrasion by the ringed stent-grafts, and the enlarged suture holes in this device (**C4**). (**A1**,**B1**,**C1**) Gross observations; (**A2**,**B2**,**C2**) Observation in light microscopy with low magnification (10×); (**A3**,**B3**,**C3**) Observation in light microscopy with high magnification (40×); (**A4**,**B4**,**C4**) Observation in SEM (100×).

Figure 6 summarizes the results of 48 h of the accelerated twist fatigue test. Again, the Z-stent supported fabrics were more severely abraded than those supported with ringed stents (Figure 6, A1, B1 and C1). Broken sutures were only found in the stent-grafts supported by the 35° Z-stents. (Figure 6, A2, B2 and C2). The fabrics of the stent-grafts were more abraded by the Z-stents than by the ringed stents (Figure 6, A3, B3 and C3). According to the SEM results, the damage from testing after 48 h was similar to that after 24 h. The fatigue resistance of the fabrics supported by the ringed stents was better than that of the Z-stents, but the suture holes of the ringed stents device were found to be considerably enlarged after removing the sutures and stents. The 45° Z-stents caused less damage than the 35° Z-stents (Figure 6, A4, B4 and C4).

**Figure 7:**
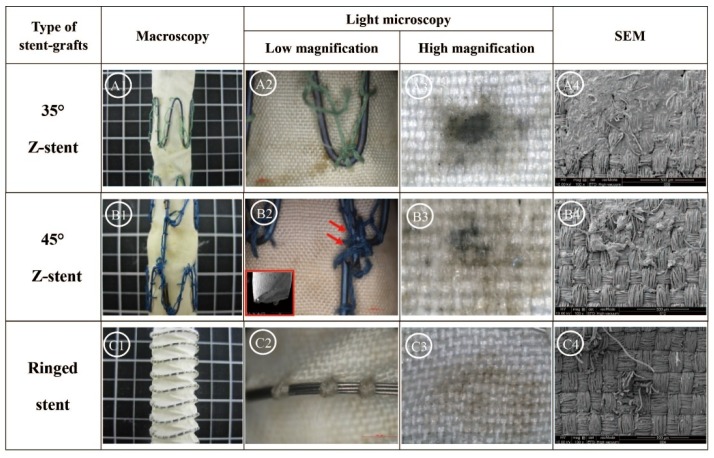
Twist tests of stent-grafts after 168 h. The stent-grafts supported by Z-stents are damaged to various levels. No holes are observed but a vertical strut of S5 is broken and numerous sutures are broken. The abrasion of the fabric is visible in the Z-stent devices, whereas it is well preserved in the ringed stent-grafts. (**A1**,**B1**,**C1**) Gross observations; (**A2**,**B2**,**C2**) Observation in light microscopy with low magnifications; (**A3**,**B3**,**C3**) Observation in light microscopy with high magnification (40×); (**A4**,**B4**,**C4**) Observation in SEM (100×).

Figure 7 summarizes the results of 168 h of the accelerated twist fatigue test on each stent-graft design. The stent-grafts supported by Z-stents were seriously damaged (Figure 7, A1, B1 and C1), but the ring-supported stent-grafts were not. Although no fabric holes were observed, most of the sutures on both Z-stent supported stent-grafts were ruptured, and the vertical strut of the S5 device (45° Z-stent) was fractured (Figure 7, A2, B2 and C2). Due to the repeated twisting, the fabrics surrounding the apexes of the Z-stents and ringed stents became black (Figure 7, A3, B3 and C3). The fibers of stent-grafts supported by Z-stents were extensively damaged. There were no obvious abrasion marks on fabrics of stent-grafts supported by ringed stents (Figure 7, A4, B4 and C4).

### 2.3. Textile Analyses of Fabrics

**Figure 8:**
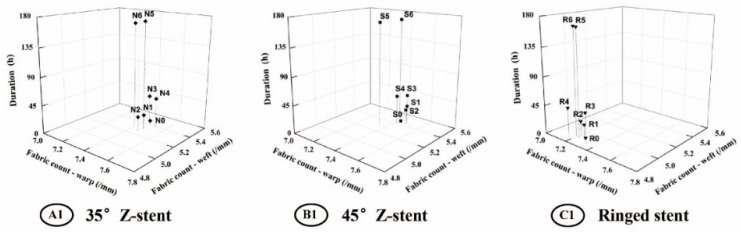
Fabric counts of the fabrics of the stent-grafts. Both the warp and the weft counts decrease slightly after twisting simulation. (**A1**) 35° Z-stent; (**B1**) 45° Z-stent; (**C1**) Ringed stent.

**Figure 9:**
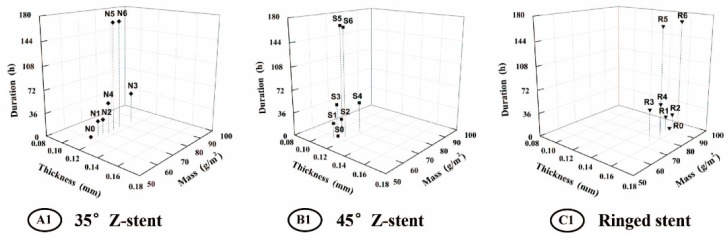
Thickness and mass of the fabrics of the stent-grafts. (**A1**) 35° Z-stent; (**B1**) 45° Z-stent; (**C1**) Ringed stent.

**Figure 10:**
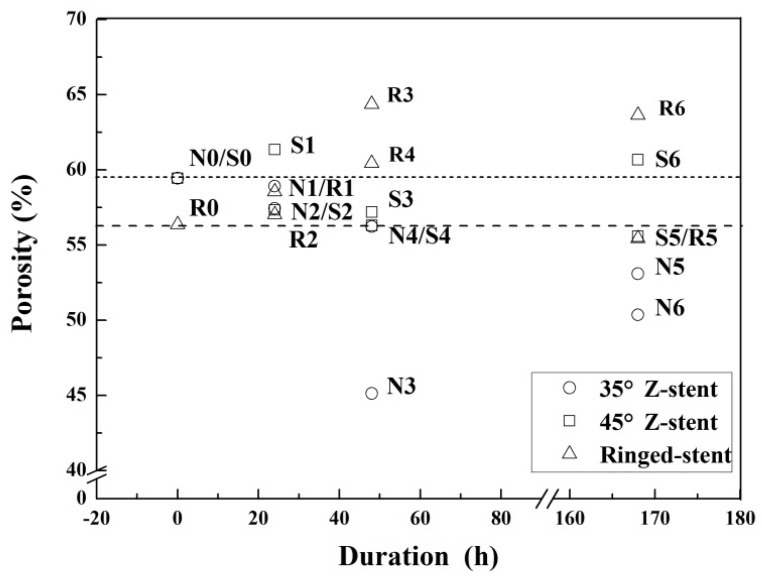
Porosity of the fabrics of the stent-grafts.

**Figure 11:**
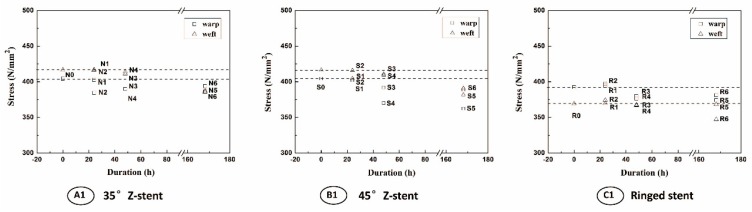
Tensile strength of fibers from the stent-grafts after different test durations. (**A1**) 35° Z-stent; (**B1**) 45° Z-stent; (**C1**) Ringed stent.

**Figure 12:**
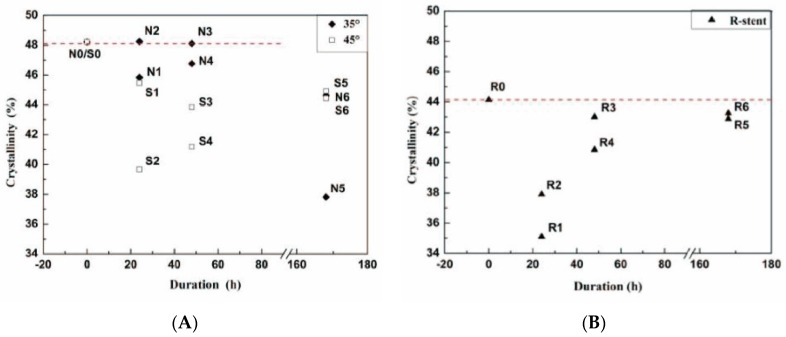
Crystallinity of the polyester in the stent-grafts. After 168 h of twisting, crystallinity was decreased by up to 10% in the Z-stent devices, compared to less than 2% in the ringed–stent devices. (**A**) 35° and 45° Z-stent; (**B**) Ringed stent.

The parameters of all fabrics investigated in this study are presented in Table 1. All devices had a plain fabric structure, which is used in most commercial stent-grafts. After the twist fatigue test, the fabric counts of all fabrics decreased slightly compared to the control (Figure 8). The thicknesses of the fabrics supported by Z-stents ranged from 0.107 to 0.117 mm. The thickness of the fabrics supported by ringed stents varied from 0.153 to 0.160 mm (Figure 9). The mass of fabrics increased slightly after the test. The increase in fabric mass was greater for fabrics supported by Z-stents than by ringed stents (Figure 9). The porosity of fabrics supported by ringed stents increased with the duration of the test while those supported by Z-stents presented a tendency to decrease (Figure 10). Compared to their controls, the fiber tensile strength declined both in warp and weft direction after twisting. The fiber tensile strength decreased less for fabrics supported by ringed stents than by Z-stents (Figure 11). The crystallinity of the fabrics tested for 168 h was lower than the control fabrics. But the crystallinity of the samples tested for 24 and 48 h was even lower than the samples tested for 168 h. The crystallinity decreased more for fabrics supported by 35° Z-stents decreased than by 45° Z-stents or ringed stents (Figure 12).

**Table 1:** Evolution of the structural parameters and crystallinity of the fabrics.

Device	No.	Duration (h)	Fabric Count (mm)		Thickness (mm)	Mass (g/m^2^)	Porosity (%)
			Warp	Weft			
Z-stent	N0/S0	0	7.42 ± 0.05	5.46 ± 0.02	0.110 ± 0.002	61.33 ± 2.31	59.45
35°	N1	24	7.48 ± 0.11	5.30 ± 0.06	0.113 ± 0.003	64.00 ± 4.00	58.92
	N2		7.47 ± 0.14	5.25 ± 0.03	0.114 ± 0.005	66.67 ± 8.33	57.44
	N3	48	7.45 ± 0.09	5.41 ± 0.02	0.113 ± 0.004	85.33 ± 4.62	45.13
	N4		7.52 ± 0.09	5.39 ± 0.06	0.115 ± 0.002	69.33 ± 8.33	56.24
	N5	168	7.39 ± 0.08	5.42 ± 0.03	0.113 ± 0.003	73.33 ± 2.31	53.10
	N6		7.39 ± 0.10	5.31 ± 0.06	0.113 ± 0.003	77.33 ± 8.33	50.36
45°	S1	24	7.44 ± 0.14	5.51 ± 0.09	0.110 ± 0.003	58.67 ± 6.11	61.35
	S2		7.48 ± 0.15	5.44 ± 0.02	0.109 ± 0.007	64.00 ± 4.00	57.34
	S3	48	7.48 ± 0.08	5.45 ± 0.02	0.106 ± 0.005	62.67 ± 4.62	57.20
	S4		7.43 ± 0.16	5.39 ± 0.11	0.117 ± 0.008	70.67 ± 6.11	56.31
	S5	168	7.35 ± 0.03	5.30 ± 0.08	0.107 ± 0.003	65.33 ± 6.11	55.29
	S6		7.37 ± 0.10	5.45 ± 0.01	0.116 ± 0.004	62.67 ± 4.62	60.68
R-stent	R0	0	7.26 ± 0.18	4.92 ± 0.05	0.153 ± 0.009	81.33 ± 10.07	56.37
	R1	24	7.28 ± 0.15	4.89 ± 0.10	0.154 ± 0.007	84.00 ± 4.00	58.57
	R2		7.22 ± 0.05	4.92 ± 0.01	0.155 ± 0.026	85.33 ± 8.33	57.04
	R3	48	7.33 ± 0.21	4.86 ± 0.14	0.155 ± 0.010	78.67 ± 8.33	64.38
	R4		7.18 ± 0.17	4.85 ± 0.06	0.154 ± 0.016	85.33 ± 6.11	60.45
	R5	168	7.24 ± 0.14	4.89 ± 0.11	0.156 ± 0.008	85.33 ± 12.22	55.44
	R6		7.20 ± 0.05	4.90 ± 0.07	0.160 ± 0.006	84.00 ± 4.00	63.65

## 3. Discussion

### 3.1. Potential Endotesion Issue

Device-related adverse events have been regularly decreasing as manufacturers have introduced new generations of devices [28,30]. Endovascular surgery has gained a considerable maturity since the pioneering work of Volodos [47,48,49]. Multi-center trials and follow-up evaluations have extended endovascular techniques to increasingly hostile anatomies as well as elderly and frail patients. Complications from device migration have been diminished by the addition of reinforced double-ringed stents, hooks, and collars [50,51,52,53,54,55]. However, new failure modes have been observed as surgeons have treated patients with more complex anatomies. Evidence of buckling and twisting has been observed in explanted devices [56,57,58]. We have previously addressed the issue of buckling. Buckling could result in major damage to the fabrics and fractures to the stents, seriously impairing the durability of devices [37]. Twisting did not show the same dramatic failures in this study, and probably does not cause major fabric perforation. However, twisting may cause yarn shifting, possibly allowing blood percolation through the fabric. Percolation of low volumes of blood through multiple sites of damage to the fabric may explain the endotension phenomenon [59,60,61,62,63,64,65,66,67,68,69,70,71,72,73,74,75,76,77,78,79,80,81,82,83,84].

### 3.2. The Fabric Structures and Stent Shape of Stent-Grafts

The fabric structures, as quantified by the fabric count, thickness and mass, did not show major differences following the twisting tests. However, it is thought that after the twist fatigue test, the mass of fabrics in fact declines, due to damage to the fabrics and sutures. A small amount of colloid from the rubber sleeve was found bonded inside the fabrics after testing. The added colloid mass compensates for the fabric removed by damage, resulting in the mass measured for the fatigue tested devices being similar to that of the untested controls. The Z-stents were more abrasive than the ringed stents, resulting in more damage to the fabrics as well as more colloid transferred to the Z-stent supported stent-grafts. Another material (such as polyurethane tubing) may not have transferred to the devices being tested.

The porosity measured in the ringed-stent devices increased after twist fatigue tests, due to the enlargement of the voids in the fabrics and the suture holes. However, the measured porosity of the Z-stent devices decreased after the testing because the voids of the fabrics were filled with colloid. 

Twist fatigue tests decreased the tensile strength of fibers from fabrics used in the devices. Fabrics supported by Z-stents had a greater decrement of fiber tensile strength, as a result of these stents abrading the fabric more than the ringed stents.

After fatigue testing, the fabric crystallinity was consistent with the fiber tensile strength of the respective stent-graft designs. The crystallinity declined at 24 and 48 h, due to the damage to the fiber macromolecule chains. Such damage to the microstructure could decrease the strength of the fabrics. The subsequent increase in crystallinity at 128 h could be from the reorientation of the macromolecule chains. Thus, the strength of fabrics may have recovered slightly. On the whole, the mechanical condition of the fabrics declined after the twist fatigue test and the fabrics were seriously abraded in the vicinity of the Z-stents, and especially at the tip of the apexes. The fabrics supported by the ringed stents were intact after 168 h of the twisting test, while the stent-grafts supported by Z-stents were damaged to various extents. Furthermore, the fabric abrasion of the 45° Z-stent devices was less severe than that of the 35° Z-stent. This suggests that more blunted stent apexes cause less abrasion to the fabrics. 

The fabric of stent-grafts can be understood as alternating regions of fabric supported by a stent and fabric without support. Most twisting of the fabric in the Z-stent devices is in the unsupported fabric. The stents inhibit twisting in the band of supported fabric. Since the ringed stent devices have more fabric that is not directly supported, there is more fabric to accommodate twisting. At 168 h, the abrasion of the fabrics supported by ringed stents was mild compared to those supported by the Z-stents.

### 3.3. The Impact of Twisting on Hemodynamics

**Figure 13:**
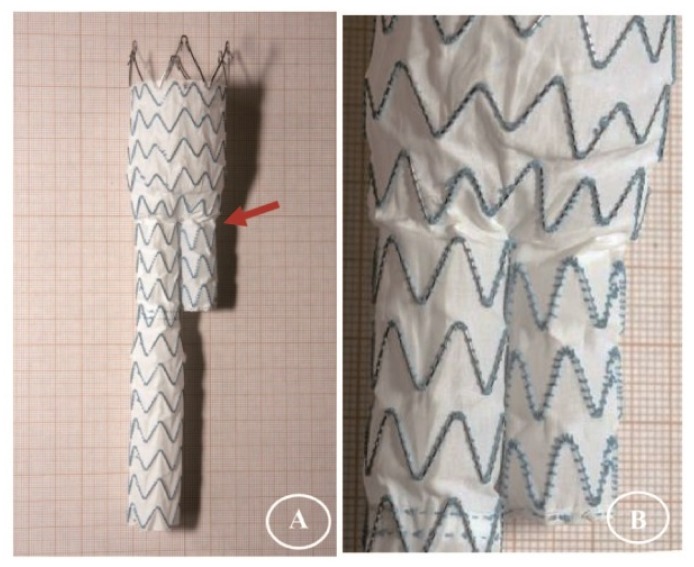
Endurant stent-graft. The fabric of the body and the limbs are supported by M-stents (**A**). Twisting might still exist in the vicinity of the crotch at the bifurcation (**A**: arrow), which is shown magnified (**B**).

**Figure 14:**
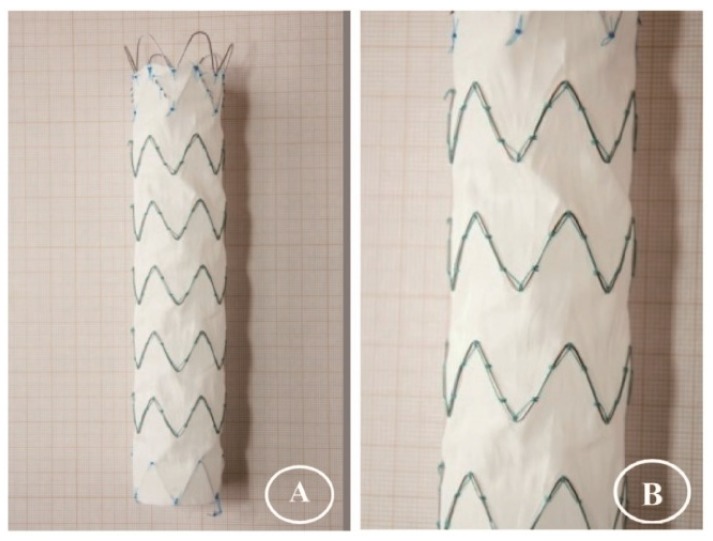
Thin wall Cook LP. Compared to the bifurcated stent-graft, the straight tubular device is less vulnerable to damage from twisting (**A**). Though the moderate angles of the Z-stents are less likely to damage the fabric, twisting may not be completely eliminated in cases with poor device deployment or complex anatomy. This could cause concentration of twisting in the unsupported fabric between two adjacent Z-stents (**B**).

The impact of twisting on hemodynamics should be considered. Thrombosis is a hazard that cannot be ignored [85,86,87,88,89,90,91]. New twisting resistant devices are now commercially available, such as Endurant and Cook LP. But the potential risk has not been fully eliminated. In particular, bifurcated prostheses may be more vulnerable to damage from twisting (Figure 13) than straight grafts (Figure 14).

## 4. Materials and Methods

### 4.1. Selection of Stent-Grafts

**Figure 15:**
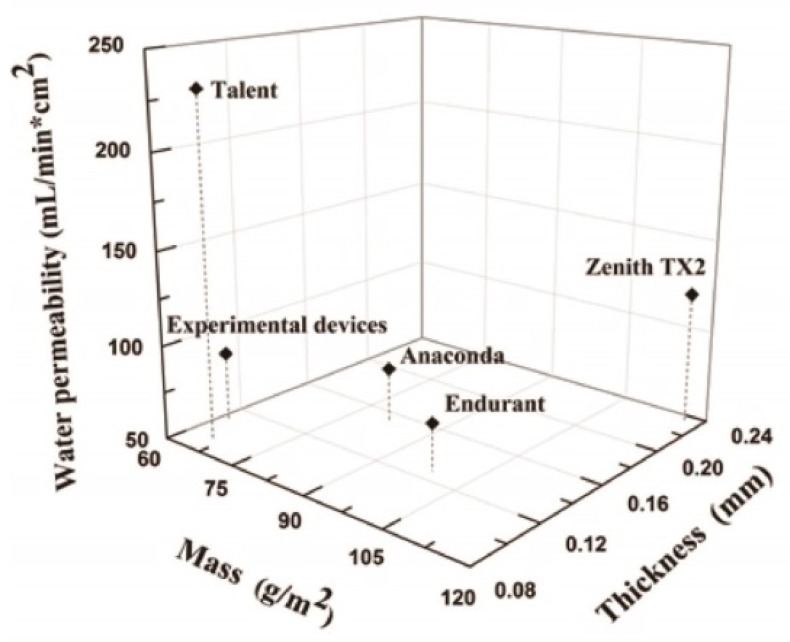
Comparison of the experimental devices to commercially available stent-grafts. They have water permeability similar to those of multifilament devices, whereas the Talent stent-graft has the highest permeability.

The experimental, Z-stent supported stent-grafts were assembled at the College of Textiles, Donghua University (Shanghai, China). They consisted of a seamless fabric tube (10 cm long and 1.0 cm in diameter) of woven polyester (Guangci Co., Shanghai, China) and Nitinol Z-stents (Longhe Metal Materials Processing Factory, Nanjing, China). The characteristics of this woven fabric were similar to those of commercially available devices (Figure 15; Table 2 and Table 3). The fabric tubes were supported by three Z-stents, sewn externally using 80 stitches of a braided multifilament polyester 5-0 suture (Jinghuan Medical Company, Shanghai, China). A vertical strut linked the three stents. Each stent was made of a 0.5 mm diameter Nitinol monofilament wire, as in the Talent stent-graft. Each stent had five apexes with strut angles at 35° for one device design and 45° for the other device design. In the 35° design, the length of each Nitinol wire was 5.4 cm, the height of each Z-stent was 1.2 cm, and the distance between two consecutive stents was 2.1 cm. In the 45° design, the length of the Nitinol wire was 4.6 cm, the height of each Z-stent was 0.8 cm, and the distance between two consecutive stents was 1.9 cm.

**Table 2:** Characteristics of fabrics in selected commercially available stent-grafts.

Graft	Fabric Structure	Fabric Count (mm)		Number of Filaments		Filament Diameter	
		Warp	Weft	Warp	Weft	Warp	Weft
Experimental devices (Guangci)	Plain	7.42 ± 0.05	5.46 ± 0.02	48	16	11.97 ± 1.21	15.31 ± 1.37
Anaconda (Vascutek)	Plain	7.26 ± 0.18	4.92 ± 0.05	27	54	12.87 ± 0.64	12.87 ± 0.64
Talent (Medtronic)	4/4 twill	20.41 ± 0.12	26.30 ± 0.13	1	1	36.30 ± 1.05	35.71 ± 1.48
Endurant (Medtronic)	Plain	9.32 ± 0.14	5.70 ± 0.06	27	27	17.56 ± 0.52	18.14 ± 0.27
Zenith TX2 (Cook)	Fancy warp-backed ^1^	5.93 ± 0.07 ^2^	5.28 ± 0.05	54	54	15.63 ± 0.70	15.44 ± 1.60

Notes: ^1^ plain + double warp; ^2^ plain: 1.98 ± 0.07; double warp: 3.95 ± 0.07.

**Table 3:** Fabric properties.

Graft	Thickness (mm)	Mass (g/m^2^)	Crystallinity (%)	Water permeability (mL/min∙cm^2^)
Experimental devices (Guangci)	0.110 ± 0.002	61.33 ± 2.31	48.21	86.6
Anaconda (Vascutek)	0.153 ± 0.009	81.33 ± 10.07	44.14	79.1
Talent(Medtronic)	0.091 ± 0.003	65.91 ± 9.14	48.56	231.5
Endurant (Medtronic)	0.127 ± 0.006	98.67 ± 8.33	32.06	75.4
Zenith TX2 (Cook)	0.234 ± 0.010	117.35 ± 4.37	34.62	121.0

**Figure 16:**
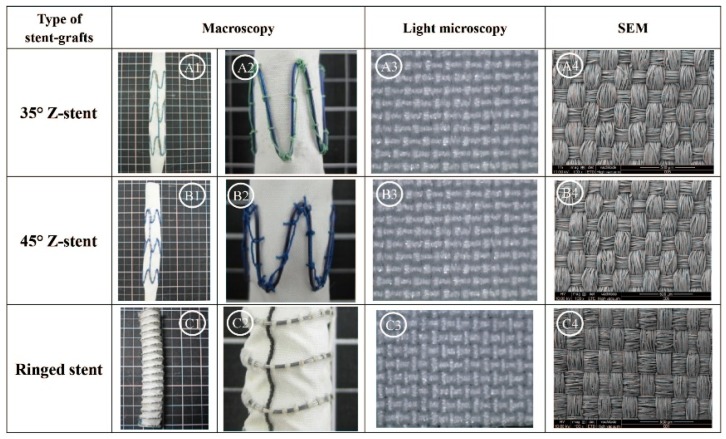
Z-stent *vs.* ringed stent. The two Z-stents were assembled in our laboratory. The seamless woven fabric tube (1.0 cm diameter and 10 cm long) was supported by Z-stents, held together by a vertical strut and sutured externally to the fabric. The apex angles of the two Z-stents were 35° (**A1**,**A2**,**A3**,**A4**) and 45° (**B1**,**B2**,**B3**,**B4**), respectively. The ringed stent-graft was a commercially available device (Anaconda), externally supported by 27 ringed stents, individually sutured to the seamless woven fabric tube (1.0 cm diameter and 10 cm long) (**C1**,**C2**,**C3**,**C4**).

The limbs of the Anaconda stent-grafts (Vascutek, a Terumo Company, Inchinnan, Scotland, UK) were also selected for this study. The Anaconda stent-grafts were supported with ringed stents. The Anaconda limbs were 1.0 cm in diameter and 10 cm long, with 0.6 cm between consecutive stents. The Anaconda design was a plain-woven polyester fabric tube supported by 27 consecutive multifilament Nitinol rings, which were sewn together by braided polyester ligatures. The Nitinol wire diameter was 0.12 mm, and each ring consisted of six turns, with the ends of the ring closed by a metallic crimp hidden under the sutures. Each ringed stent required 23 stitches to fix it to the graft tube. Both the Z-stent and the ringed-stent devices are illustrated in Figure 16.

### 4.2. Twisting Fatigue Experiment

#### 4.2.1. Fatigue Machine 

**Figure 17:**
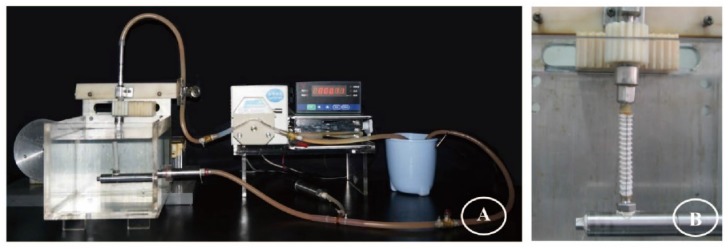
The fatigue machine developed at Donghua University simulates the twisting observed for stent-grafts *in vivo*. It mimics the pulsatile dynamics within human tortuous iliac arteries (**A**). The device is attached by its first extremity to a fixed vertical metallic pipe. The second extremity is attached to an upper metallic vertical pipe, which can be rotated clockwise and anticlockwise to cause repeated twisting of the stent-graft (**B**). Distilled water is circulated from the reservoir to the vertical metallic tube through an electronic pulsatile pump. After passing through the stent-graft (lined with an impervious latex membrane), the pressure and volumetric rate of the water flow is regulated and returned to the reservoir. The temperature in the basin is maintained at 37 °C.

The fatigue machine was developed at Donghua University to mimic the pulsatile dynamics of the blood flow within human tortuous iliac arteries (Figure 17). The unique feature of the device was an oscillating-twist mechanism. One extremity of the stent-graft was fixed whereas the opposite one was rotated clockwise and anticlockwise causing repeated twist of the stent-graft. Distilled water at 37 °C filled a tank to submerge the stent-graft and was circulated through the device by an electronic peristaltic pump (DDBT-201, Shanghai Wuxiang Instrument Co., Shanghai, China). A thin rubber sleeve was fitted inside the stent-graft to ensure stable pressure and prevent the distilled water from permeating through the fabric of the stent-graft.

#### 4.2.2. Fatigue Conditions

**Figure 18:**
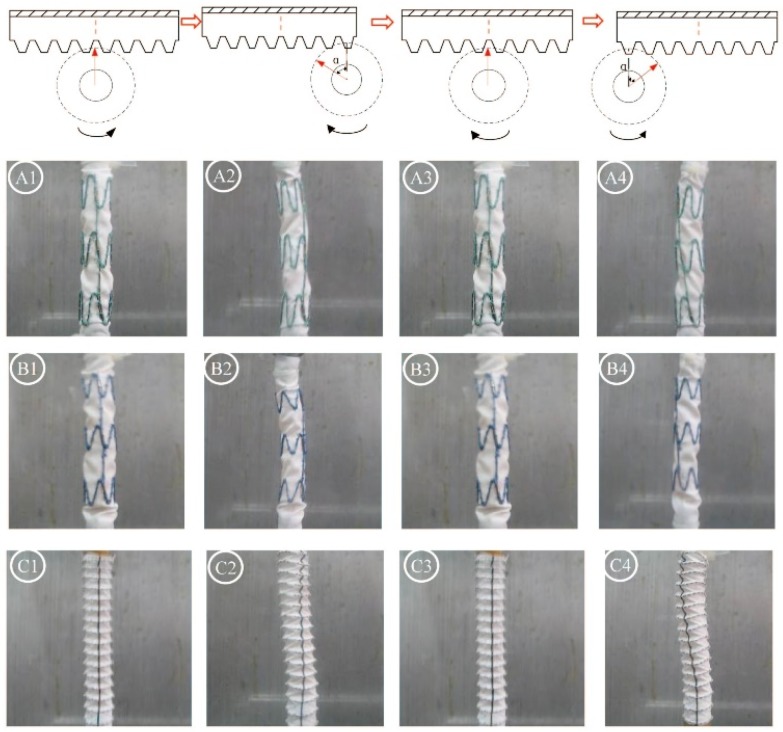
The twist process applied to the stent-grafts. The 35° Z-stent (**A1**), 45° Z-device (**B1**) and the ringed device (**C1**) behave dramatically different. When the gear rotates towards the right, one end of the device is turned anticlockwise (**A2**,**B2**,**C2**) to some twist angle. When the gear rotated towards the left, the devices are returned to their original state (**A3**,**B3**,**C3**) firstly, and then to an anticlockwise twist angle (**A4**,**B4**,**C4**). The Z-stent devices were more severely distorted than the ringed devices, and thus the distilled water flow was more impaired.

A pulsation frequency of 10 Hz (1 to 1.67 Hz in humans) and pressure of 48 kPa (16 kPa or 120 mmHg in humans) was applied to provide accelerated fatigue conditions. The twisting was through an angle of 60 degrees at 1 Hz. Figure 18 illustrates the stent-grafts through the procedure of the twist-fatigue experiment.

The tests were scheduled for durations of 24 h, 48 h, and 168 h (1, 2, and 7 days). Two specimens were tested for each duration. Tests were immediately stopped if the polyester fabric ruptured or the Nitinol wires fractured. Devices without any loading were used as controls (0 h). The number of pulsations and twists are given in Table 4.

### 4.3. Analyses of Stent-Grafs

#### 4.3.1. Gross Observations

Each device was examined and photographed with a digital camera (Sony DSC F707, 5 Megapixel, Sony China Co., Shanghai, China). The presence of stent fracture, fabric holes, and ligature ruptures were inspected through digitally magnified images.

**Table 4:** Number of pulsations and twists.

Conditions	0 h	24 h	48 h	168 h
Pulsations	0	8.64 × 10^5^	1.73 × 10^6^	6.048 × 10^6^
Twists	0	8.64 × 10^4^	1.73 × 10^5^	6.048 × 10^5^

#### 4.3.2. Observation in Light Microscopy

The devices were inspected at 40× magnification with a light compound microscope PXS8-T (Cewi Photoelectric Technology Co., Shanghai, China) fitted with a CCD camera (Digital Sight DS-Fi1, Nikon Imaging China Sales Co., Shanghai, China).

#### 4.3.3. Observation in SEM

The sutures were carefully cut, allowing the stents to be removed from the fabrics before the SEM observation. The devices were observed in a Jeol JSM-5600LV environmental scanning electron microscope (Jeol Ltd., Tokyo, Japan) at a 10 kV accelerating voltage. Special attention was paid to the instances of yarn shifting, holes, fabric structure distortions, and filament damage.

### 4.4. Textile Analyses of Fabrics

#### 4.4.1. Fabric Structure and Fabric Count

Fabric structure and fabric count (the number of ends and picks in the woven fabric) were determined with a light compound microscope at 40× magnification. MB-Ruler (a software package) was used with the microscope to get the number of ends and picks within 1 mm, considering the scale of the image. Twenty different locations on each fabric specimen were measured to calculate the mean and standard deviation.

#### 4.4.2. Thickness

A thickness gauge CH-12.7-BTSX (Shanghai Liuling Instrument Plant, Shanghai, China) with a division of 0.001 mm was employed to measure the fabric thickness. The diameter of the columniform gauge head was 5 mm, and thus the area of the presser foot was 19.63 mm^2^. The testing pressure was 22 ± 5 kPa (44 ± 10 g). Each specimen was cleaned, then measured at ten random locations with the average of these reported.

#### 4.4.3. Mass

The masses of the fabric specimens were measured with an electronic analytical balance (FA2004, Shanghai Liangping Instrument Co., Shanghai, China) with 0.1 mg resolution. Specimens of 1 × 1 cm^2^ were selected at three locations from each cleaned fabric. Each specimen was measured five times, with the average reported. The mass per unit area (g/m^2^) of the fabric specimens was calculated.

#### 4.4.4. Porosity

The porosity (P) of the fabric (*i.e.*, the volume of the void space as a percentage of the total volume of the fabric) was calculated as: P (%) = 100 × (1 − 1000M/Atρ), where M is the total mass (g); A is the total area (mm^2^); t is the thickness (mm) of the fabric; and ρ is the density of the polyester fiber (1.38 g/cm^3^).

#### 4.4.5. Fiber tensile strength 

The tensile strength of fibers was performed with a single fiber electronic tensile strength tester (Model LLY-06GE, Laizhou Electronic Instrument Co., Laizhou, Shangdong, China), using a method modified from the GB14337-2008 standard. The fibers were carefully extracted from fabrics after the twisting fatigue tests. Fibers were kept in a constant temperature (20 °C) and humidity (65%) room for 24 h before the tensile tests. The initial distance between the clamps on the fibers was 10 mm and the tensile speed was 20 mm/min. The pre-tension of the PET fibers is 0.25 cN. From each fabric specimen, 30 fibers were extracted and tested from the warp and weft direction, respectively.

#### 4.4.6. X-ray Diffraction (XRD)

An XRD D/Max-2550 PC (RIGAKU Co., Ltd., Tokyo, Japan) was used to measure the degree of crystallinity of fabrics, which describes the molecular structure of the polyester filaments. Damage to these molecular structures would alter the mechanical characteristics of the fabric.

## 5. Conclusions

Twisting in stent-grafts could cause device-related adverse events and efforts must be pursued to dampen the height and the angle of the Z-stents. Sharp angles of stent apexes appear to be responsible for major damage to the fabric from twisting of the stent-grafts. The various manufacturers are currently developing innovative devices that are likely to be more resistant to twisting.
